# Association between human T cell leukemia virus type-1 (HTLV-1) infection and advanced periodontitis in relation to atherosclerosis among elderly Japanese: a cross-sectional study

**DOI:** 10.1186/s12199-019-0836-2

**Published:** 2019-12-28

**Authors:** Yuji Shimizu, Hirotomo Yamanashi, Masayasu Kitamura, Reiko Furugen, Takahiro Iwasaki, Hideki Fukuda, Hideaki Hayashida, Koji Kawasaki, Kairi Kiyoura, Shin-Ya Kawashiri, Toshiyuki Saito, Atsushi Kawakami, Takahiro Maeda

**Affiliations:** 1 Department of Community Medicine, Nagasaki University Graduate School of Biomedical Sciences, Nagasaki-shi, Sakamoto 1-12-4, Nagasaki, 852-8523 Japan; 2 Department of Cardiovascular Disease Prevention, Osaka Center for Cancer and Cardiovascular Disease Prevention, Osaka, Japan; 3 Department of General Medicine, Nagasaki University Hospital, Nagasaki, Japan; 4 Department of Oral Health, Nagasaki University Graduate School of Biomedical Sciences, Nagasaki, Japan; 5 Department of Community Oral Health, School of Dentistry, Asahi University, Gifu, Japan; 6 Community Medical Network Center, Nagasaki University Hospital, Nagasaki, Japan; 7 Department of Immunology and Rheumatology, Nagasaki University Graduate School of Biomedical Sciences, Nagasaki, Japan; 8 Department of Island and Community Medicine, Nagasaki University Graduate School of Biomedical Sciences, Nagasaki, Japan

**Keywords:** Atherosclerosis, Elderly, HTLV-1, Periodontitis

## Abstract

**Background:**

Human T cell leukemia virus type-1 (HTLV-1) stimulates inflammation activity. Our previous study revealed a positive association between asymptomatic HTLV-1 infection and advanced periodontitis among elderly Japanese individuals with low levels of hematopoietic activity (reflected by reticulocyte levels). Since low hematopoietic activity has been correlated with low-grade inflammation and low-grade inflammation is associated with atherosclerosis, the status of atherosclerosis could, in turn, determine the nature of this association.

**Methods:**

To this end, a cross-sectional study of 907 elderly Japanese individuals (aged 60–99 years), who had participated in dental health check-up during the period 2016–2018, was conducted. Advanced periodontitis was defined as periodontal pocket ≥ 6.0 mm.

**Results:**

Among the study population, 295 (32.5%) were found to have atherosclerosis defined as a carotid intima-media thickness (CIMT) of ≥ 1.1 mm. HTLV-1 infection was positively associated with advanced periodontitis in participants with atherosclerosis, but no significant associations were observed among the participants without atherosclerosis. The known risk factors’ (including reticulocyte and CIMT) adjusted odds ratio (OR) and 95% confidence interval (CI) of advanced periodontitis were OR 2.01 and 95% CI 1.06–3.81 for participants with atherosclerosis and OR 0.61 and 95% CI 0.34–1.12 for participants without atherosclerosis.

**Conclusion:**

This study found a significant association between HTLV-1 infection and advanced periodontitis among elderly Japanese with atherosclerosis. However, this association is absent in individuals without atherosclerosis, suggesting that atherosclerosis might act as a determinant in the association between HTLV-1 infection and advanced periodontitis among elderly Japanese.

## Background

Although human T cell leukemia virus type-1 (HTLV-1), which is the earliest recognized human retrovirus, has been shown to be a causative agent of adult T cell leukemia/lymphoma [1], as well as associated with myelopathy/tropical spastic paraparesis, sensorimotor polyneuropathy, and optic neuritis [2], the majority of carriers remain asymptomatic throughout their lives [3–5].

However, we have previously reported the positive association of asymptomatic HTLV-1 infection to advanced periodontitis in elderly subjects with low hematopoietic activity (and low reticulocyte levels) [6]. Although HTLV-1 enhances inflammation activity [7, 8], the factor that activates HTLV-1 is unknown. However, the influence of HTLV-1 infection should be stronger in participants with inflammation than in those without inflammation. Severe cases of chronic periodontitis were associated with endothelial (microvascular) dysfunction [9, 10]. Therefore, the presence of inflammation associated with endothelial dysfunction might act as a determinant factor in the association between HTLV-1 infection and advanced periodontitis. As low hematopoietic activity is associated with low-grade inflammation [11–14], elderly subjects with low hematopoietic activity could assume an important role in the association between HTLV-1 infection and advanced periodontitis [6].

In addition, low-grade inflammation is associated with atherosclerosis [15] and subsequent disruption of microcirculation [16]. Therefore, asymptomatic HTLV-1 infection could be positively associated with advanced periodontitis in subjects diagnosed with developing atherosclerosis.

Since periodontitis is a major cause of tooth loss which results in a significant decrease in the quality of life of elderly people [17] and HTLV-1 is known to be a common retrovirus [18], clarifying the association between HTLV-1 infection and advanced periodontitis in relation to atherosclerosis is important in preserving the quality of life among elderly population. However, we are unaware of any study that has clarified the associations between HTLV-1 infection and advanced periodontitis in relation to the status of atherosclerosis.

To evaluate the association between HTLV-1 infection and advanced periodontitis in relation to atherosclerosis status, we conducted a cross-sectional study involving 907 elderly people aged 60–99 years who participated in annual dental health check-ups during 2016–2018.

## Methods

### Study population

The study population comprised of 1925 elderly Japanese (702 men and 1223 women) aged 60–99 years from Goto City in the western part of Japan who attended annual health check-ups during 2016–2018 conducted by the local government and directed by the Ministry of Health, Labor and Welfare in Japan and who underwent oral assessment.

Participants without data on carotid intima-media thickness (CIMT) (*n* = 4) and those without any remaining teeth (*n* = 920) were excluded. Advanced periodontitis is diagnosed as the maximum probing pocket depth for each gingiva. Incidentally, decayed teeth are the most frequent reason for teeth extraction and subsequent periodontal diseases in Japan [19]. Therefore, a small number of participants with normal teeth might induce a detection bias for periodontitis. On this basis, subjects with 1–4 remaining teeth (*n* = 90), as well as people with missing HTLV-1 data (*n* = 3) and missing reticulocyte data (*n* = 1), were excluded. The remaining participants comprised of 353 men and 554 women with a mean age of 72.1 (standard deviation [SD] 7.0) for men and 71.3 (SD 6.8) for women were enrolled in the study.

### Data collection and laboratory measurements

Trained interviewers obtained information on smoking status. Body weight and height in bare feet and light closing were measured by using an automatic body composition analyzer (BF-220; Tanita, Tokyo, Japan). Body mass index (BMI) was calculated as weight (kg)/height (m)^2^.

Blood samples were collected with siliconized, sodium fluoride, and EDTA-2K containing tubes. All measurements were made following standard laboratory procedures at SRL, Inc. (Tokyo, Japan). Samples from the siliconized tube were used to measure high-density lipoprotein (HDL)-cholesterol and triglycerides, and sample from the sodium fluoride tube was used to measure glycated hemoglobin (HbA1c). Red blood cells (RBC) and reticulocytes in the samples collected in EDTA-2K tubes were measured. Reticulocyte levels were determined using the following formula: reticulocytes (× 10^4^ cells/μL) = (reticulocytes, ‰) × RBC (× 10^4^ cells/μL)/1000. The calculated values were 5.358 ± 2.141 [× 10^4^ cells/μL] and 5.047 ± 1.774 [× 10^4^ cells/μL] for male and female participants, respectively.

### Measurement of carotid intima media-thickness

Experienced vascular technicians measured carotid intima media-thickness (CIMT) using a LOGIQ Book XP with a 10-MHz transducer (GE Healthcare, Milwaukee, WI, USA). Maximum values for the left and right common carotid arteries of the CIMT were calculated with automated digital edge-detection software (Intimascope; MediaCross, Tokyo, Japan) following a protocol that has been described in detail elsewhere [20]. The higher values of right and left CIMT not including plaque measurements were then calculated, and the maximum CIMT value was used for analysis. Since a previous study reported a CIMT value of < 1.1 mm as normal [21], we defined samples with a CIMT value of ≥ 1.1 mm as atherosclerotic.

### Measurement of human T cell leukemia virus type-1 (HTLV-1)

To detect human T cell leukemia virus type-1 (HTLV-1), a chemiluminescent enzyme immunoassay (CLEIA) kit (Fujirebio Inc., Tokyo, Japan) was used.

### Oral examination

Trained dentists (*n* = 6) performed periodontal examination by using a modified method from the Third National Health and Nutrition Examination Survey (USA) [22]. Probing pocket depth was measured using a periodontal probe at the mesiobuccal and mild-buccal sites for all present teeth excluding the third molars. Prior to the start of this study, all examiners were trained and calibrated using a chart, periodontal models, and volunteers at the Nagasaki University Hospital.

### Statistical analysis

Atherosclerosis was diagnosed as having CIMT ≥ 1.1 mm, and the study population was stratified into those with and without atherosclerosis. Atherosclerosis status-specific differences in mean values or proportions of variables by HTLV-1 status were analyzed. A trend test was performed with analysis of variance (ANOVA) for continuous values, and chi-squared test was used for determining differences in proportions. Advanced periodontitis was diagnosed as a probing pocket depth of ≥ 6 mm.

Logistic regression models were used to calculate odds ratios (ORs) and 95% confidence interval (CIs) to determine the association between HTLV-1 infection and advanced periodontitis. Since atherosclerosis might act as a determinant factor on the association between HTLV-1 infection and advanced periodontitis as we hypothesized in the present study, participants were stratified according to their atherosclerosis status.

Periodontitis risk factors that have a direct influence on the intra-oral environment, such as smoking [23], and the number of remaining teeth were regarded as confounding factors. While no significant correlation between caries (decayed teeth) and periodontitis was observed in previous studies, both conditions have a common etiological factor [24, 25]. Further, decayed teeth are the most frequent reason for teeth extraction and subsequent periodontal diseases in Japan [19]. Hence, we also included decayed teeth as a confounding factor in our analysis. CIMT and reticulocytes were included in the analysis as confounding factors due to their possible mechanistic role in the development of atherosclerosis. Obesity defined by BMI and diabetes are known risk factors for periodontitis [26, 27]. Moreover, while no association between dyslipidemia and periodontitis has been reported [27], hypo-HDL cholesterol and hyper-triglycerides could be associated with periodontitis [28]. Thus, we included BMI, HbA1c, HDL cholesterol, and triglycerides as confounding factors in our logistic regression models. Further, since medication for diabetes [29] and hyperlipidemia [30] might influence the severity of periodontitis, we included these as confounding factors.

Three different models were used to adjust for confounding factors. Model 1 was only adjusted for sex and age. For model 2, we included other potential confounding factors, namely BMI (kg/m^2^), smoking status (non-smoker or current smoker), number of remaining teeth, decayed teeth (presence, absence), HDL cholesterol (mg/dL), triglycerides (mg/dL), HbA1c (%), reticulocyte (cells/μL), and CIMT (mm). Model 3 was further adjusted for the use of glucose-lowering medication (yes, no) and antihyperlipidemic medication (yes, no).

In addition, we evaluated the influence of atherosclerosis on the association between HTLV-1 infection and advanced periodontitis by logistic regression analysis.

Furthermore, we also performed sex-specific analyses to determine the association between HTLV-1 infection and advanced periodontitis according to the status of atherosclerosis.

As reticulocyte levels have an ambivalent association with hypertension (systolic blood pressure ≥ 140 mmHg and/or diastolic blood pressure ≥ 90 mmHg and/or having used antihypertensive medication) and atherosclerosis in the elderly [31], we also conducted analyses of the relationship between reticulocytes (tertile values) and hypertension and that of reticulocytes (tertile values) and atherosclerosis using a sex- and age-adjusted model.

All statistical analyses were performed with SAS system for Windows (version 9.4: SAS Inc., Cary, NC). A *p* value of < 0.05 was considered statistically significant.

## Results

Of the study population, 295 and 197 were diagnosed as having atherosclerosis and advanced periodontitis, respectively.

### Characteristics of the study population

Among the participants without atherosclerosis, people who were HTLV-1 positive were older compared to those who were HTLV-1 negative. Among the participants with atherosclerosis, HTLV-1 positive individuals had significantly lower triglycerides and HbA1c levels than people who were HTLV-1 negative (Table 1).

**Table 1:** Characteristics of the study population

	Atherosclerosis					
	(−)			(+)		
	HTLV-1 infection		*p*	HTLV-1 infection		*p*
	(−)	(+)		(−)	(+)	
No. of subjects	512	100		232	63	
Men, %	36.3	31.0	0.309	47.8	39.7	0.251
Age, year	70.1 ± 6.5	72.0 ± 6.6	0.008	73.7 ± 6.8	75.6 ± 6.9	0.055
Body mass index (BMI), kg/m^2^	22.7 ± 3.2	23.4 ± 3.4	0.043	23.4 ± 3.4	22.8 ± 3.1	0.151
Serum HDL cholesterol, mg/dL	63 ± 15	60 ± 16	0.062	61.3 ± 13.7	63.8 ± 15.6	0.223
Serum triglycerides, mg/dL	103 ± 52	111 ± 61	0.188	99 ± 46	84 ± 30	0.013
Hemoglobin A1c (HbA1c), %	5.7 ± 0.5	5.8 ± 0.5	0.060	5.9 ± 0.6	5.7 ± 0.4	0.007
Glucose lowering medication use, %	3.9	6.0	0.343	10.8	31.1	0.064
Antihyperlipidemic medication use, %	19.5	20.0	0.914	25.9	20.6	0.396
Current smoker, %	6.3	6.0	0.925	6.0	6.3	0.927
No. of remaining teeth	22.0 ± 6.4	22.0 ± 6.0	0.999	20.3 ± 6.6	20.2 ± 7.4	0.923
Individuals with decayed teeth, %	29.1	31.0	0.704	30.6	25.4	0.423
CIMT, mm	0.83 ± 0.10	0.84 ± 0.10	0.405	1.17 ± 0.18	1.15 ± 0.22	0.553
Reticulocyte, × 10^4^ cells/μL	5.169 ± 1.806	5.280 ± 1.740	0.574	5.071 ± 1.663	5.331 ± 3.510	0.405

Values: mean ± 1 standard deviation (SD). *CIMT* Carotid Intima-Media Thickness. Atherosclerosis is defined as carotid intima-media thickness (CIMT) ≥ 1.1 mm.

Participants with advanced periodontitis show significantly higher values of BMI and decayed teeth (Table 2).

**Table 2:** Characteristics of the study population

	Advanced periodontitis		
	(−)	(+)	*p*
No. of subjects	710	197	
Men, %	36.6	47.2	0.007
Age, year	71.5 ± 7.0	72.3 ± 6.3	0.147
HTLV-1 infection, %	17.7	18.8	0.738
Body mass index (BMI), kg/m^2^	22.8 ± 3.2	23.6 ± 3.4	0.002
Serum HDL cholesterol, mg/dL	62 ± 15	61 ± 15	0.454
Serum triglycerides, mg/dL	101 ± 50	101 ± 53	0.908
Hemoglobin A1c (HbA1c), %	5.8 ± 0.5	5.8 ± 0.5	0.953
Glucose lowering medication use, %	5.5	7.1	0.394
Antihyperlipidemic medication use, %	21.3	21.3	0.987
Current smoker, %	5.4	9.1	0.051
No. of remaining teeth	21.6 ± 6.6	20.8 ± 6.3	0.123
Individuals with decayed teeth, %	27.0	38.1	0.003
CIMT, mm	0.94 ± 0.21	0.96 ± 0.20	0.200
Reticulocyte, × 10^4^ cells/μL	5.152 ± 1.771	5.223 ± 2.422	0.650

Values: mean ± 1 standard deviation (SD). *CIMT* Carotid Intima-Media Thickness. Advanced periodontitis is defined as periodontal pocket ≥ 6.0mm

### Association between advanced periodontitis and HTLV-1 infection among total participants

From the logistic regression models, the ORs and 95% CIs for the correlation of advanced periodontitis to HTLV-1 infections among all participants are shown in Table 3. No significant association between HTLV-1 infection and advanced periodontitis were observed.

**Table 3:** Odds ratios (ORs) and 95% confidence intervals (CIs) for advanced periodontitis in relation to human T cell leukemia virus-1 (HTLV-1) infections

	HTLV-1 infection		*p*
	(−)	(+)	
No. of subjects	744	163	
No. of case (%)	160 (21.5)	37 (22.7)	
Model 1	1.00	1.07 (0.71, 1.61)	0.764
Model 2	1.00	1.05 (0.69, 1.60)	0.814
Model 3	1.00	1.06 (0.70, 1.60)	0.800

Advanced periodontitis is defined as periodontal pocket ≥ 6.0 mm. Atherosclerosis is defined as carotid intima-media thickness (CIMT) ≥ 1.1 mm. Model 1: adjusted only for sex and age. Model 2: adjusted further for smoking status (never, former, current), number of remaining teeth, status of decayed teeth (presence, absence), body mass index (BMI), HDL-cholesterol, triglycerides, hemoglobin A1c (HbA1c), reticulocyte count, and CIMT. Model 3: adjusted further for antihyerlipidemic medication use (yes, no) and glucose-lowering medication use (yes, no)

### Association between advanced periodontitis and HTLV-1 infection stratified by atherosclerosis status

Atherosclerosis-status-specific ORs and 95% CIs for advanced periodontitis in relation to HTLV-1 infections are presented in Table 4. No significant association between HTLV-1 and advanced periodontitis was observed for participants without atherosclerosis. However, in the participants with atherosclerosis, being positive for HTLV-1 infection was associated with a greater likelihood of advanced periodontitis. An investigation into the effects of the associations between HTLV-1 infection and atherosclerosis on advanced periodontitis revealed significant interactions.

**Table 4:** Odds ratios (ORs) and 95% confidence intervals (CIs) for advanced periodontitis in relation to human T cell leukemia virus type-1 (HTLV-1) infections by status of atherosclerosis

	Atherosclerosis						Interaction
	(−)			(+)			
	HTLV-1 infection		*p*	HTLV-1 infection		*p*	*p*
	(−)	(+)		(−)	(+)		
No. of subjects	512	100		232	63		
No. of case (%)	108 (21.1)	15 (15.0)		52 (22.4)	22 (34.9)		
Model 1	1.00	0.64 (0.35, 1.15)	0.135	1.00	2.02 (1.09, 3.73)	0.026	0.015
Model 2	1.00	0.62 (0.34, 1.13)	0.116	1.00	2.00 (1.05, 3.78)	0.034	0.011
Model 3	1.00	0.61 (0.34, 1.12)	0.113	1.00	2.01 (1.06, 3.81)	0.033	0.013

Advanced periodontitis is defined as periodontal pocket ≥ 6.0 mm. Atherosclerosis is defined as carotid intima-media thickness (CIMT) ≥ 1.1 mm. Model 1: adjusted only for sex and age. Model 2: adjusted further for smoking status (never, former, current), number remaining teeth, status of decayed teeth (presence, absence), body mass index (BMI), HDL-cholesterol, triglycerides, hemoglobin A1c (HbA1c), reticulocyte count, and CIMT. Model 3: adjusted further for antihyerlipidemic medication use (yes, no) and glucose-lowering medication use (yes, no)

### Sex-specific association between advanced periodontitis and HTLV-1 infection stratified by atherosclerosis status

Sex-specific ORs and 95% CIs for advanced periodontitis regarding HTLV-1 infection according to the status of atherosclerosis are shown in Table 5. Even the statistical value could not reach a significant level, and essentially the same associations were observed for men and women.

**Table 5:** Sex-specific odds ratios (OR) and 95% confidence intervals (CIs) for advanced periodontitis in relation to human T cell leukemia virus-1 (HTLV-1) infections by status of atherosclerosis

	Atherosclerosis						Interaction
	(−)			(+)			
	HTLV-1 infection		*p*	HTLV-1 infection		*p*	*p*
	(−)	(+)		(−)	(+)		
Men							
No. of subjects	186	31		111	25		
No. of case (%)	45 (24.2)	7 (22.6)		29 (26.1)	12 (48.0)		
Model 1	1.00	0.88 (0.35, 2.18)	0.774	1.00	2.68 (1.09, 6.96)	0.032	0.106
Model 2	1.00	0.79 (0.30, 2.05)	0.624	1.00	3.39 (1.24, 9.24)	0.017	0.071
Model 3	1.00	0.81 (0.31, 2.11)	0.662	1.00	3.76 (1.34, 10.56)	0.012	0.058
Women							
No of subjects	326	69		121	38		
No of case (%)	63 (19.3)	8 (11.6)		23 (19.0)	10 (26.3)		
Model 1	1.00	0.51 (0.23, 1.14)	0.100	1.00	1.56 (0.66, 3.70)	0.309	0.083
Model 2	1.00	0.51 (0.23, 1.16)	0.108	1.00	1.46 (0.57, 3.69)	0.430	0.111
Model 3	1.00	0.50 (0.22, 1.15)	0.102	1.00	1.48 (0.58, 3.76)	0.413	0.090

Advanced periodontitis is defined as periodontal pocket ≥ 6.0 mm. Atherosclerosis is defined as carotid intima-media thickness (CIMT) ≥ 1.1 mm. Model 1: adjusted only for age. Model 2: adjusted further for smoking status (never, former, current), number of remaining teeth, status of decayed teeth (presence, absence), body mass index (BMI), HDL cholesterol, triglycerides, hemoglobin A1c (HbA1c), reticulocyte count, and CIMT. Model 3: adjusted further for antihyperlipidemic medication use (yes, no) and glucose-lowering medication use (yes, no)

### Association between reticulocytes and hypertension, and reticulocytes and atherosclerosis

By using a sex- and age-adjusted model, analysis between tertiles of reticulocytes and hypertension, and between tertiles of reticulocytes and atherosclerosis was performed. Even the power did not reach significant value; reticulocyte count is positive tendency with hypertension and inverse tendency with atherosclerosis. Using the lowest tertile of reticulocyte count (< 4.390 × 10^4^ cells/μL for men and < 4.140 × 10^4^ cells/μL for women) as a reference group, the adjusted ORs for hypertension and atherosclerosis were 1.08 (0.77, 1.50) and 0.93 (0.66, 1.32) for the second tertiles of reticulocyte count (4.390–5.724 × 10^4^cells/μL for men and 4.140–5.551 × 10^4^cells/μL for women) and 1.21 (0.86, 1.70) and 0.89 (0.62, 1.27) for the highest tertile of reticulocyte count (5.725 × 10^4^cells/μL ≤ for men and 5.552 × 10^4^cells/μL ≤ for women) (not shown in table).

Sensitivity analysis shows that excluding participants with less than 10 remaining teeth, as reported in previous studies [6, 9], yields trends similar to our major observations.

## Discussion

In this study, we found no significant association between HTLV-1 infection and advanced periodontitis in elderly people without atherosclerosis. However, in elderly people with atherosclerosis, HTLV-1 infection was associated with an increased likelihood of advanced periodontitis.

Previously, we reported that HTLV-1 infection was positively related to advanced periodontitis among elderly participants with lower hematopoietic activity (lower reticulocyte count) but not among elderly participants with higher hematopoietic activity (higher reticulocyte count) [6]. Lower hematopoietic activity is the main cause of anemia in the elderly, and unexpected anemia in the elderly is characterized by the incidence of low-grade inflammation [11]. In particular, elderly subjects suffering from anemia because of lower hematopoietic activity might be more susceptible to low-grade inflammation. Since erythrocytes possess antioxidant capacity [12] and activation of erythropoiesis can cause reduction in oxidative stress [13], anemia increases oxidative stress. Low-grade inflammation, shown to be well connected with oxidative stress [14], is also associated with periodontitis [32]. Therefore, the observed positive association between HTLV-1 infection and advanced periodontitis among elderly subjects with low reticulocyte counts could be due to the presence of low-grade inflammation.

Although in the present study the positive association between HTLV-1 infection and advanced periodontitis was observed only among participants with atherosclerosis, the biological characteristics of HTLV-1 might explain the finding. HTLV-1 possesses a characteristic of inflammation inducer since the trans-activator protein of Tax of HTLV-1 enhances inflammation [7, 8]. The influence of HTLV-1 infection should be stronger in participants with inflammation than in those without inflammation. Since low-grade chronic inflammation causes progression of atherosclerosis [15] and disruption of microcirculation [16], the influence of HTLV-1 on endothelial (microvascular) dysfunction should be stronger for participants with atherosclerosis than that for participants without atherosclerosis. Further, severe cases of chronic periodontitis were recently reported to be associated with endothelial (microvascular) dysfunction [9, 10]. Therefore, low-grade inflammation, but not hematopoietic activity itself and/or atherosclerosis, might be the culprit behind the reported data. This could explain the invariance of our major observations, even after necessary adjustments for reticulocyte and CIMT were incorporated in the data analysis.

However, as shown in Table 1, among participants with atherosclerosis, we found no significant differences in CIMT values between the HTLV-1 (−) and HTLV (+) groups. Among the participants with atherosclerosis, HTLV-1 (+) could have lower classical cardiovascular risk factors such as low HDL-cholesterol, high triglycerides, and high HbA1c than HTLV (−) which might also act as confounding factors. This suggests that despite clinical characteristics of HTLV-1 (+) subjects showing a lower risk of atherosclerosis progression than that of HTLV-1 (−) subjects, the former might develop atherosclerosis. Further investigations with information on gingival microcirculation are necessary.

Furthermore, even the statistical power in this study could not reach a significant value among participants without atherosclerosis, compared to participants without HTLV-1 infection. Also, participants with HTLV-1 infection showed lower ORs in advanced periodontitis (Table 4) with higher CIMT values and higher reticulocyte values (Table 1).

Low-grade inflammation also induces hypertension [33]. Previously, we reported that height is an indicator of the capacity of vascular repair in elderly men [34], especially hypertensive men. In addition to that, elderly men with a short stature might have lower hematopoietic capacity evaluated by reticulocyte but have higher vascular remodeling activity than those with a high stature [35, 36]. These studies have indicated that erythropoietic activity, estimated according to reticulocyte levels, could be an indicator of endothelial maintenance capacity and activity among elderly individuals.

A previous study with 2098 elderly participants reported that reticulocyte count was positively associated with hypertension, whereas it was inversely associated with atherosclerosis [31]. In our additional analyses, even the power could not reach significant value because of the small number of participants (*n* = 907), and reticulocyte count showed essentially the same tendency for hypertension and atherosclerosis. Hypertension is well known as a significant factor in endothelium injury, and atherosclerosis is a result of aggressive endothelial repair. Therefore, even a higher hematopoietic activity should have a higher tissue-repairing activity such as reduction of a marker of oxidative stress [13] and higher hematopoietic activity is also induced by more severe tissue injury (increased oxidative stress) [37, 38]. These associations indicate that the low level of reticulocytes itself indicates that low-grade inflammation tissue injury, such as advanced periodontitis, might elevate reticulocyte count. Therefore, no significant differences in reticulocyte counts between participants with and without advanced periodontitis were observed in this study (Table 2). Even though aging reduces this type of response activity resulting in tissue injury, it could not induce sufficient reticulocyte production. Therefore, anemia in the elderly is associated with low-grade inflammation [11]. Further investigation with larger samples and wider age ranges is necessary to clarify those associations.

Aging is a process that increases vascular aging-related inflammation [39]. Age-related decline of bone marrow activity (the productivity of hematopoietic stem cells) is associated with endothelial repair deficiency which results in lower CIMT [40, 41] and reticulocyte values [42]. Therefore, higher reticulocyte and CIMT values among elderly participants without atherosclerosis could have a beneficial influence on endothelium maintenance to some extent by promoting appropriate endothelial repair activity. Then, the inflammation inducer characteristics of HTLV-1 [7, 8] could have a beneficial influence on endothelial maintenance to some extent among elderly participants without atherosclerosis by stimulating bone marrow activity (the productivity of hematopoietic stem cells). Further investigation with information on hematopoietic stem cells is necessary.

Therefore, HTLV-1 infection that stimulates inflammation activity [7, 8] could have a beneficial association with advanced periodontitis among participants without atherosclerosis, but an unfavorable association with advanced periodontitis among participants with atherosclerosis. This might result in no significant association between HTLV-1 infection and advanced periodontitis among elderly participants (Table 3).

The clinical implication for the present study is that, even in non-symptomatic status, HTLV-1 infection could be associated with advanced periodontitis and atherosclerosis status might influence the association between HTLV-1 infection and advanced periodontitis among elderly people.

Potential limitations of this study warrant consideration. Although the age-related endothelial dysfunction may influence the association between HTLV-1 infection and advanced periodontitis, no data on the evaluation of endothelial function was available. Thus, further analyses that include endothelial function-related data such as flow-mediated dilation (FMD) will be necessary. This study did not include confirmatory tests for detection of HTLV-1. In spite of that, we believe that the influence of HTLV-1 was limited, especially since our previous study, using real-time reverse transcription-polymerase chain reaction with the hydrolysis probe and western blotting assays, showed only a small false positive rate (1.2%) [43]. In addition, we have no information on the hygiene status of these samples. Previous studies have reported that the number of remaining teeth among the elderly Japanese depends on the type of dental visit [44]. Furthermore, tooth loss leads to reduced nutrient intake [45]. Therefore, we have used data on the number of remaining teeth as a confounding factor in our analysis. Further, we do not have the setup ready for evaluating potentially crucial cases of low-grade inflammation. The statistical value of sex-specific analyses is limited in the present study because of the small number of participants. However, essentially, the same associations were observed for men and women. Additionally, because this was a cross-sectional study, causal relationships cannot be established.

## Conclusion

In conclusion, we found HTLV-1 infection to be positively associated with advanced periodontitis among elderly participants with atherosclerosis but not among those without atherosclerosis. Therefore, atherosclerosis might be a crucial factor that determines the association of HTLV-1 infection with advanced periodontitis among elderly Japanese people. These results were an efficient tool to clarify the role of the possible mechanism for the association between HTLV-1 and microcirculation.

## Data Availability

The datasets generated during and/or analyzed during the current study are not publicly available due to ethical consideration but are available from the corresponding author on reasonable request.

## Abbreviations

ANOVA: Analysis of variance
BMI: Body mass index
CIMT: Carotid intima-media thickness
CIs: Confidence intervals
CLEIA: Chemiluminescent enzyme immunoassay
FMD: Flow-mediated dilation
HDL: High-density lipoprotein
HTLT-1: Human T cell leukemia virus type-1
ORs: Odds ratios
RBC: Red blood cell
